# COVID-19 pandemic and stability of stock market—A sectoral approach

**DOI:** 10.1371/journal.pone.0250938

**Published:** 2021-05-20

**Authors:** Michał Buszko, Witold Orzeszko, Marcin Stawarz

**Affiliations:** 1 Department of Financial Management, Faculty of Economic Sciences and Management, Nicolaus Copernicus University in Torun, Torun, Poland; 2 Department of Applied Informatics and Mathematics in Economics, Faculty of Economic Sciences and Management, Nicolaus Copernicus University in Torun, Torun, Poland; The Bucharest University of Economic Studies, ROMANIA

## Abstract

The COVID-19 pandemic seems to be the most important phenomenon observed from March 2020 in virtually all countries of the world. The necessity to prevent the spread of COVID-19 and keep health care systems efficient resulted in the forced, drastic limitation of economic activity. Many service sectors were hit particularly hard with this but industry and agriculture were also affected. In particular, the pandemic substantially influenced financial markets and we can observe that some markets or instruments vary in stability since they have been affected in the different degree. In the paper, we present the problem of stability of stock markets during the COVID-19 pandemic. Due to the low number of works related to CEE countries during the pandemic, we analyze the Warsaw Stock Exchange, which is one of the most important markets in the CEE. Our main goal was to find how various industries represented by stock market indices have reacted to the COVID-19 shock and consequently which sectors turned out to keep stability and remained resistant to the pandemic. In our investigation, we use two clustering methods: the *K*-means and the Ward techniques with the criterion of maximizing the silhouette coefficient and six indicators describing stability in terms of profitability, volume, overbought/oversold conditions and volatility. The results of the research present that during the pandemic it was possible to identify 5 clusters of sector indices in the short term and 4 in the medium term. We found that the composition of the clusters is quite stable over time and that none of the obtained clusters can be univocally considered the most or the least stable taking into account all the analyzed indicators. However, we showed that the obtained clusters have different stability origins, i.e. they vary from each other in terms of the investigated indicators of stability.

## 1. Introduction

The COVID-19 pandemic had a significant impact on the socio-economic life of most countries in the world. The virus has the potential to influence in a destructive way individuals, businesses, industries and entire economies [1]. Its appearance, in principle, meant a significant burden and reorganization of the health service, and the need to provide additional disinfection and hygiene measures, but its global scope is expected to be the most influencing economic and social event for decades [2]. The problem of the COVID-19 virus has been its rapid spread, which resulted from airborne transmission and intensive use of public transport, including intercontinental flights. The necessity to prevent the failure of health care systems and counteracting the effects of COVID-19, which poses a direct threat to the health and life of citizens, resulted in the forced and often drastic limitation of economic activity. In fact, the pandemic brought global economic activity to a sudden halt in the first half of 2020 [3]. The sectors hit particularly strongly were services, including tourism and hoteling, retail trade, education, cultural activities, restaurants, galleries, gyms, hairdressers, taxis, expos, sporting events and personal services characterized by direct contact between people [4]. Land and air transport, as well as entities directly related to it, including airports, also suffered severely. Especially, in the last sector, the demand is expected to be highly affected not only in the medium term, but also in the long term [5] as the dynamics of pandemic spread is strictly linked with the airlines system [6]. Moreover, Liu et al. [7] provided systematic analysis on the dynamics and dimensions of the unprecedented decline in the public transit demand due to the pandemic. The need for isolation and the inability to conduct production, commercial or service activities on the current terms resulted in the emergence of disruptions in production and supply, as well as the breakdown of logistics chains. The problem was also employees’ infections, which made it impossible to conduct business in an undisturbed manner. The pandemic led also to a substantial fall in energy demand and global CO_2_ emissions [3]. Where the specific nature of the activity allowed it, the COVID-19 pandemic contributed to a change in the organization and work model of many entities, causing their decentralization, forcing greater flexibility of operation and starting the transformation towards remote work but also influencing internal relations, employee adjustment and human resources management [8]. The issue of respecting human rights in such conditions was also raised [9].

The period of the pandemic is undoubtedly a turning point in the activities of many sectors, as well as for the directions of development of the entire economies, definitely changing the economic realities more than the previous crisis of 2007–2009 [10]. It can be treated as a specific demand and supply shock, the source of which is the lockdown of the real economy and disruptions in service, trade and production activities resulting from sanitary and epidemic reasons. The pandemic and its effects in finance are being compared to the previous global financial crisis 2007–2009. Wojcik and Ioannou [11] find that the previous crisis is rather referred as the North Atlantic crisis which was spread around the world through international financial and economic relations, but the COVID-19 pandemic is truly global and directly affecting practically all countries because of traveling. In contrary to the 2007–2009 crisis, the pandemic crisis has not been initiated in the financial sector but its severity in the real sphere has transmitted it into financial sector and then reversely again to the production, trading and services. In some industries, it has undoubtedly caused a significant change in the business model or affect a change in the incomes and costs structure. The consequences of changes and transformations in individual sectors are currently difficult to predict, as it is unknown how long the pandemic will ultimately last and what its costs will be. Undoubtedly, there will be new challenges in the area of computerization, logistics, personnel management, real estate management, cybersecurity and broadly understood health protection.

The COVID-19 pandemic is a new phenomenon, therefore the studies on it are still new but quickly expanding. The issue of COVID-19 was taken up primarily in medicine and pharmacy [12–16], but shortly afterwards it started to be a subject of research in many other disciplines such as health sciences [17, 18], psychology [19, 20] or physics and mathematics [21]. In the field of economic sciences, especially finance, the achievements regarding COVID-19 are getting more and more common and refer mostly to the consequences of the lockdowns. It has been relatively short time to observe the phenomenon and obtain data reflecting its impact on financial statements but works related to various aspects of COVID-19 in finance and capital markets have been published. The financial issues during the pandemic have been a subject of investigations so far in scope of insurance [22], banking [23–25] or financial system [26]. One can also find results of research performed in other financial aspects linked with COVID-19, such as those presenting the influence of the dynamics of the panic level due to COVID-19 shock onto movements of the exchange rates [27]. The influence of the pandemic onto alternative investments such as cryptocurrencies is also becoming important area of the research in finance [28–32].

A growing number of papers linking COVID-19 and finance relates to stock markets. The literature in this scope contains works published even before the outbreak of the pandemic but suitable for explaining investor behaviors in the COVID period as well as works completed during the pandemic. In the first group, one may find papers addressing the issues of contagion [33], spillovers between markets during shocks [34] as well as the impact of bad news on the time-varying betas [35]. In the second, there are papers related to issues of dependencies between global factors and markets [36] or to links between individual stock market reactions and severity of the outbreak of pandemic in various countries [37]. Moreover, one can list some other works, e.g. related to pricings of stock during the pandemic. Singh [38] found that investors become more attentive to corporate fundamentals and ESG that support the long-run sustainability of firms during turbulence. Fundamental aspects of investments were also pointed out by Mirza et al. [39] who found that social entrepreneurship investment funds outperformed their counterparts during the outbreak of the pandemic. In the field of stock pricing and price tendencies, Shehzad et al. [40] found that pandemic has influenced the variance of the US, Germany, and Italy’s stock markets stronger than the global financial crisis. Against this background, Narayan et al. [41] and Phan & Narayan [42] found positive effects of lockdowns, travel bans, and economic stimulus packages onto stock markets, and Sharif et al. [43] found that in the US the pandemic outbreak has a greater effect on the geopolitical risk and economic uncertainty than the stock market itself.

The review of the literature shows a relatively low number of works related to the CEE countries where the pandemic has also been strongly affecting the financial markets. The reason behind such phenomenon may be the relatively large fragmentation of local capital markets in the CEE region, their small capitalization in most of the countries, as well as the overall lower financialization of the economies compared to highly developed countries. In this scope the investigation of Topcu and Gulal [44] reveals that the negative impact of the pandemic on emerging stock markets was stronger in Asia than in Europe and has gradually fallen and begun to taper off by mid-April. The authors also point that in emerging markets the size of stimulus packages provided by the governments matter in offsetting the effects of the pandemic. Other research related to exchange rates and stock market behavior of the Visegrad countries of CEE during the pandemic shows a significant and negative link between the Visegrad stock market indices and the COVID-19 spread [2].

Apart from not many works related to the pandemic in CEE, a largely missing part in the current research is the influence of COVID-19 onto specific sectors of economies. Some results of sectoral affection can be found in Wojcik and Ioannou [11] pointing the relatively lowest downgrading of the health care and consumer goods and the highest in energy, financial and industrial sectors. Other evaluation of stock market under the pandemic terms was performed by Haroon and Rizvi based on sectoral indices for US from Dow Jones [45]. They showed that panic induced by COVID-19 related news was positively associated with volatilities in indices of several industrial sectors such as transportation, automobiles & components, energy and travel & leisure. Similar results on sectoral returns for the Australian stock market were obtained by Najdu and Ranjeeni [46]. Another analysis was conducted by Mazur et al. [47] who examined the effect of COVID-19 on stock market behavior of S&P1500 companies at the industry-level, including their stock pricing and volatility. The authors found that during the stock market crash stocks in healthcare, food, natural gas, and software sectors performed abnormally well whereas companies from crude oil industry, real estate, entertainment and hospitality sectors were the worst performers. Moreover, the authors found that loser’s stocks had more asymmetric movements and exhibited high volatility that correlated negatively with stock returns. Looking at the sectoral susceptibility to COVID-19 pandemic, Akhtaruzzaman et al. [48] found that financial firms play more important role in financial contagion than nonfinancial firms. Apart from that, some sectoral evaluation was also performed based on Chinese companies and China stock market, including He et al. [49], Gu et al. [50] and Xiong et al. [51]. All the above-mentioned research were focused mainly on stock market pricing or volatility analysis but not directly on complex evaluation of the stability.

The review of the research on the influence of the pandemic and finance reveals a relatively low number of investigations related to stability of financial markets after the outbreak of the pandemic COVID-19. The concept of financial stability is ambiguous and can be interpreted in many ways as well as from different perspectives, including infrastructure, institutions, instruments, markets, regulations and financial results. The review of the research concerning the financial stability proves that there are no commonly accepted definitions, models or analytical frameworks for its assessment. The complexity of the topic, as well as problems with defining and measuring financial stability was shown by Schinasi [52] and Goodhart [53] whose works lead to the conclusion that a single target variable cannot be appropriate for defining and measuring financial stability. Nonetheless, financial stability in its most simple way can be defined as the situation when the financial system allows efficient allocation of savings into investments without disruptions [54]. The stability can be also identified when the financial system is able to withstand shocks without impairing the allocation of savings, investments and the processing of payments [55]. In a more complex way, the financial stability can be defined as well-functioning components of the financial system, including financial intermediaries, institutions, markets, payments, settlement and clearing systems [56, 57]. Schinasi [52] describes financial stability in terms of ability to facilitate and enhance economic processes, manage risks, and absorb shocks. The author points out also that financial stability should be considered as continuum, i.e. changeable over time and consistent with multiple combinations of the constituent elements of finance. Apart from that, the concept of financial stability may be also defined through the prism of instability, where stability is a state of affairs when instability is unlikely to occur [58]. Financial instability means then the conditions in financial markets that harm, or threaten to harm, an economy’s performance through their impact on the working of the financial system [59]. Another approach defines instability as the degree to which shocks to the financial system are amplified and propagated across markets or across institutions [60]. The concept of financial stability has been applied to the context of smooth functioning of banks and financial system [61], monetary policy and central banking [62, 63], price stability [64–67] including emerging markets [68] or economics and economic policy [69]. In the literature, one may also find more specific areas where the concept of financial stability appears, such as renewable energy [70], digital finance [71] or shadow banking [72].

In the literature on stability of financial markets after the outbreak of the pandemic COVID-19 the significantly lesser part is devoted to stock markets. The stability of stock market can be defined as the constant (stable) propagation of systematic shocks on a stock market in normal and extreme market conditions [73]. The definition and work published by the mentioned authors have led to further investigations performed by Ayinde and Yinusa [74] for African market and Chirila and Chirila [75] for CEE countries. Linking the concept of financial stability with the behavior of stock markets in terms of the COVID-19 pandemic, we identify the gap in research, related both to sectoral evaluation of the financial stability as well as to evaluation of the markets in CEE countries during the pandemic. To reduce such gap, we decided to analyze one of the most important stock markets in the CEE region, i.e. the Warsaw Stock Exchange (WSE), which provides official sectoral classification and evaluation of sectors performance with WSE sub-indices. As on October 2020, the WSE was the largest stock market in CEE in terms of the number of companies listed (434), as well as the capitalization of domestic companies (approx. EUR 200 bn.). Additionally, since September 2018 Poland has begun to be classified in the FTSE Russel index as a highly developed country and it was included in the group of 25 countries as the first country in Central and Eastern Europe. Thus, some of the largest Polish companies were included in the indices of developed markets, and thus also in the Stoxx Europe 600 index. These facts confirm the quality and quantity of changes that have taken place on the WSE in recent years and also justify the choice of this market for our investigation.

The main goal of this paper is to assess the financial stability (resilience to shock) of 16 industries (sectors) represented by the sector indices at the WSE in the period of the outbreak of the COVID-19 pandemic. We intend to find how various industries represented by stock market indices have reacted to the COVID-19 shock during the first months of the pandemic and consequently which sectors turned out to keep stability and remained resistant to the pandemic. We see several important practical implications of such investigation, e.g., it may be used for designing and developing new investment strategies at the stock market, for restructuring investment portfolios as well as for more effective investment risk management. Thanks to it, we may better understand the behavior of various sectors and companies during the external shocks.

In our research we focus on the evaluation of the sectoral performance. The study will determine the stability criteria for the behavior of sectors before and after the onset of the pandemic, examine the similarity of sectors’ behavior by dividing them into clusters as well as their change (reclustering) in the course of the pandemic. We investigate the stability by comparing pre-pandemic and pandemic performance of indices using two time points that may be considered as the beginning of the pandemic, i.e. the first global Disease Outbreak News Report of WHO on January 5, 2020 regarding the outbreak of COVID-19 and March 12, 2020, i.e. the beginning of the lockdown in Poland and WHO declaration that the outbreak of COVID-19 is the pandemic.

As it has been noted, financial stability is a complex concept, and it cannot be measured with just one variable. Moreover, in the literature, there are no clear suggestions which indicators (variables) would be the most suitable to investigate stability of stock markets. Due to the multifaceted nature of this problem we decided to use clustering methods (namely: the *K*-means and the Ward techniques) to analyze it in a comprehensive and broad scope. Both methods have been successfully applied in the literature to investigate capital markets [76–80] and to analyze the consequences of the COVID-19 pandemic [81–84].

The contribution of the paper is primarily an evaluation of the Warsaw Stock Exchange, i.e. the largest and most developed stock market in CEE during the pandemic in scope of financial stability from the sectoral perspective. Moreover, we propose indicators which can be used for clustering in the evaluation of the stability of a stock market during shocks.

The remaining part of the paper is organized as follows: in Section 2, we describe clustering methods as well as characterize data and research process. In Section 3, we present results and discussion. The paper is finished with the conclusions.

## 2. Methods and research description

### 2.1 Clustering methods

Clustering is the unsupervised grouping of objects into classes without any *a priori* knowledge of the datasets to be analyzed [85]. The purpose of clustering is to find high-quality groups of similar objects and identify patterns in the data. The problem of clustering is to divide a given data set into clusters (groups) in such a way that data points in a cluster are more similar to each other than points in different clusters. Clustering itself should not be considered as one specific algorithm as it is a general task to be solved. This can be achieved by using different clustering methods, which vary considerably within the meaning of what constitutes a cluster and how to find them.

Most of the clustering methods can be categorized as hierarchical or partitional clustering. Algorithms of hierarchical clustering generate a cluster tree (dendrogram) by using heuristic splitting or merging techniques. By contrast, partitional methods usually require that the number of clusters and an initial clustering be specified as an input to the procedure [86, 87].

In our study we apply two clustering methods: the *K*-means and the Ward techniques. The *K*-means method [88] is a well-known partitional clustering algorithm. It determines clusters with minimal variability of the observations within each cluster, calculated using the within-cluster sum of squares:

$$\sum_{k=1}^{K}\sum_{i:x_{i}\in C_{k}}\left({\left\|x_{i}-\mu_{k}\right\|}^{2}\right),$$ (1)

where *K* is the number of clusters, *C*_*k*_ (*k* = 1,2,…,*K*) denote clusters, *μ*_*k*_ are centroids (usually described by the mean of points in the cluster *C*_*k*_). In order to indicate the optimal clustering, the iterative algorithm is performed. It starts with randomly selected (or derived from *a priori* information) initial *K* centroids. Then each point in the data set is assigned to the closest cluster (i.e. to the closest centroid), based on the distance function. Next, based on the absorbed cases, new centroids are calculated. This process is repeated until convergence is achieved [89].

The second applied technique is the Ward method of hierarchical clustering. Like other agglomerative techniques, it consists in building nested clusters by merging them successively. The result can be represented as a tree (dendrogram) which describes the hierarchy of clusters. The Ward algorithm starts with clustering where each data point forms a cluster by itself. In each step the two clusters that minimally increase within-cluster variance (i.e., the error sums of squares (1) with $\mu_{k}={\bar{x}}_{k}$) are merged. The algorithm terminates when there is only one cluster left.

To assess the results of clustering, the validation measures should be applied. Such measures can be additionally useful to choose the proper clustering method and its parameters, e.g. the assumed number of clusters [90]. One of such measure, often proposed in the literature, is the silhouette coefficient [91]. To calculate the silhouette coefficient, one needs to estimate the similarity of each point *x*_*i*_ to its own cluster compared to other clusters, using the formula:

$$s(i)=\frac{b(i)-a(i)}{max(a(i),b(i))},$$ (2)

where *a*(*i*) is the mean dissimilarity between *x*_*i*_ and the points in the cluster *C* that contains *x*_*i*_, i.e.:

$$a(i)=\frac{1}{|C|-1}\sum_{x_{j}\in C,j\neq i}d(i,j)$$ (3)

and *b*(*i*) is the smallest average dissimilarity between *x*_*i*_ and all points in any other clusters, i.e.:

$$b(i)=\underset{C_{k}\neq C}{\min}\frac{1}{\left|C_{k}\right|}\sum_{x_{j}\in C_{k}}d(i,j).$$ (4)

When |*C*| = 1, Eq (3) cannot be calculated, and it is assumed that *s*(*i*) = 0.

The measures *s*(*i*) take values from the interval 〈−1,1〉, being large (i.e., close to 1) when the point *x*_*i*_ has been assigned to an appropriate cluster. A value of 0 indicates that *x*_*i*_ is close to the decision boundary between two neighboring clusters and a negative value indicates that it might have been assigned to the wrong cluster.

By definition, *s*(*i*) measure silhouettes for single points. In turn, to assess the quality of all *K* clusters, one should calculate the average silhouette:

$$\bar{s}(K)=\frac{1}{N}\sum_{i=1}^{N}s(i),$$ (5)

where *N* is the number of points. The maximum value of $\bar{s}(K)$ (taken over all *K*) is called the silhouette coefficient (*SC*) and is the main indicator of the clustering quality. According to Kaufman and Rousseeuw [92], the silhouette coefficient can be interpreted in the following way:

- *SC*≤0.25: no substantial structure has been found,
- 0.26≤*SC*≤0.50: the structure is weak and could be artificial,
- 0.51≤*SC*≤0.70: a reasonable structure has been found,
- 0.71≤*SC*≤1: a strong structure has been found.

### 2.2 Data and research process

In order to examine the stability of the behavior of individual sectors of WSE, i.e. their resistance to the impact of the COVID-19 pandemic, we used a total of 16 indices, 14 of which were sector indices reflecting individual industries and two macro indices reflecting industries not directly included in sector indices, i.e. WIG.GAMES and WIGtech. Table 1 presents the characteristics of the investigated indices.

**Table 1:** Description of investigated indices.

Index	Number of companies	Description
WIG-banking (“WIG-banki”)	15	WIG-banking is a sub-sector index and its portfolio includes WIG constituents belonging to the ‘banking’ sector.
WIG-construction (“WIG-budownictwo”)	37	WIG-construction is a sub-sector index and its portfolio includes WIG constituents belonging to the ‘construction’ sector.
WIG-chemical (“WIG-chemia”)	5	WIG-chemical is a sub-sector index and its portfolio includes WIG constituents belonging to the ‘chemical’ sector.
WIG-energy (“WIG-energia”)	11	WIG-energy is a sub-sector index and its portfolio includes WIG constituents belonging to the ‘energy’ sector.
WIG.GAMES (“WIG.GAMES”)	5	WIG.GAMES index is calculated based on the value of portfolio of 5 most liquid companies covering game developers’ sector.
WIG-mining (“WIG-górnictwo”)	4	WIG-mining is a sub-sector index and its portfolio includes WIG constituents belonging to the ‘mining’ sector.
WIG-IT (“WIG-informatyka”)	21	WIG-IT is a sub-sector index and its portfolio includes WIG constituents belonging to the ‘IT’ sector.
WIG-pharmaceutical (“WIG-leki”)	9	WIG-pharmaceuticals is a sub-sector index and its portfolio includes WIG constituents belonging to the ‘pharmaceuticals’ sector.
WIG-media (“WIG-media”)	12	WIG-media is a sub-sector index and its portfolio includes WIG constituents belonging to the ‘media’ sector.
WIG-automobiles&parts (“WIG-motoryzacja“)	7	WIG-automobiles&parts is a sub-sector index and its portfolio includes WIG constituents belonging to the ‘automobiles and parts’ sector.
WIG-real estate (“WIG-nieruchomości”)	27	WIG-real estate is a sub-sector index and its portfolio includes WIG constituents belonging to the ‘real estate’ sector.
WIG-clothes (“WIG-odzież”)	17	WIG-clothes is a sub-sector index and its portfolio includes WIG constituents belonging to the ‘clothes & cosmetics’ sector.
WIG-oil&gas (“WIG-paliwa”)	7	WIG-oil&gas is a sub-sector index and its portfolio includes WIG constituents belonging to the ‘oil and gas’ sector.
WIG-food (“WIG-spożywczy”)	19	WIG-food is a sub-sector index and its portfolio includes WIG constituents belonging to the ‘food and drinks’ sector.
WIGtech (“WIGtech”)	44	WIGtech index is calculated based on the value of portfolio of companies covering following sectors: biotechnology, video games, IT, telecoms and new technology.
WIG-telecom (“WIG-telekomunikacja”)	4	WIG-telecom is a sub-sector index and its portfolio includes WIG constituents belonging to the ‘telecom’ sector.

Source: GPW Benchmark (https://gpwbenchmark.pl/en-notowania)

The stability analysis requires distinguishing two periods: base (pre-pandemic)–used to determine the standard behavior of the indices under study, and pandemic, in which the behavior of indices caused by the pandemic is analyzed. We assumed July 8, 2019 –January 3, 2020 (hereinafter: Period_0) as the base period, i.e., the period in which investment decision were not affected by the pandemic. As the beginning of the pandemic on the stock market, we considered two alternative time points. According to the first option, the beginning of the pandemic was assumed on January 7, 2020, i.e., the first working day after the WHO first COVID-19 Disease Outbreak News Report, and according to the second–on March 12, 2020, i.e. the first working day after the WHO statement that the COVID-19 is the pandemic and beginning of the lockdown in Poland. Moreover, we had to determine the length of the pandemic period analyzed in the study. In the case of the first time point of the beginning of the pandemic, the six-month research period was considered, i.e., January 7, 2020 –July 6, 2020 (hereinafter: Period_1). In the case of the second option, it can be seen that the period after March 12 was associated with very rapid and dynamic changes in the securities markets. At the same time, during this period, one can expect differences in the behavior of individual companies in terms of the depth and duration of these changes. For this reason, in this case, three variants of the pandemic period were considered: two weeks, 1 month and 3 months, which made it possible to identify and compare the duration of unstable reactions in individual industries. This means that in the second option, the study considered three alternative pandemic periods: March 12, 2020 –March 25, 2020 (hereinafter: Period_2a), March 12, 2020 –April 9, 2020 (hereinafter: Period_2b) and March 12, 2020 –June 10, 2020 (hereinafter: Period_2c). We will treat Period_2a and Period_2b as the short-term and Period_2c as well as Period_1 as the medium-term.

**Fig 1:**
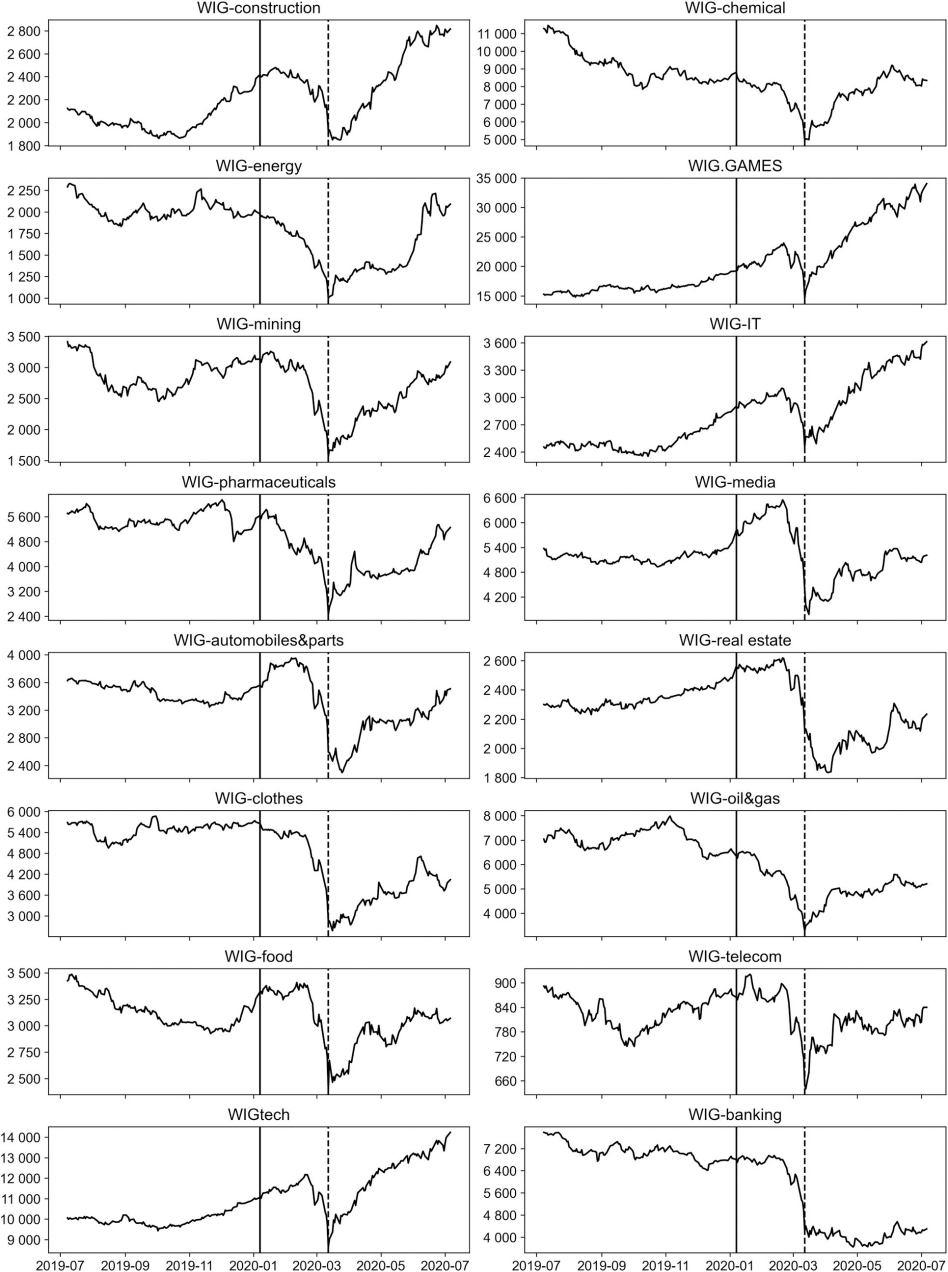
Dynamics of indices in the analyzed period.

Fig 1 shows the dynamics of all the analyzed indices before and during the pandemic period. The chart also indicates the two time points assumed in the study as the start of the pandemic. The solid line indicates January 7, 2020 and the dashed line–March 12, 2020. In the case of most indices, it can be seen that the largest decreases in the indices’ values were recorded until March 12 and the largest increases after that date.

Taking into account the issue of lacking any single variable which can be used to comprehensively define and measure stability of stock markets and their sectors (what we have underlined in the introduction), in our further investigation, we propose multiple variables as indicators of this stability. They reflect different aspects of stock market stability, including pricing, trading volume, volatility, and the attitude of investors. In our study, we used one variable describing stability in terms of profitability (price), one for volume, one for overbought/oversold conditions as well as three different measures of volatility. All of these variables reflect the difference between the pre-pandemic and pandemic periods and for the sake of comparability, most of them are presented as percentage changes.

The price stability measure (denoted by $\tilde{P}$) proposed in the study is defined by the formula:

$$\tilde{P}=\frac{\bar{P_{1}}-\bar{P_{0}}}{\bar{P_{0}}},$$ (6)

where $\bar{P_{0}}$ and $\bar{P_{1}}$ denote the average value of a given index over the base and pandemic periods, respectively. This means that $\tilde{P}$ reflects the relative change of the average price.

To assess the stability of the volatility of quotations, we used three different measures based on the standard deviation, the Parkinson estimator and the range, respectively.

The first one (denoted by $\tilde{\sigma }$) is given by the formula:

$$\tilde{\sigma }=\frac{\sigma_{1}-\sigma_{0}}{\sigma_{0}},$$ (7)

where *σ*_0_ and *σ*_1_ are the standard deviations of log-returns of a given index over the base and pandemic periods, respectively. As a result, the value $\tilde{\sigma }$ is the relative change of the standard deviation.

The second stability measure of volatility refers to the Parkinson [93] estimator of the standard deviation, expressed as:

$$\sigma_{t}^{\left(p\right)}=\sqrt{\left[ln{\left(H_{t}/L_{t})\right]}^{2}/(4ln2\right)},$$ (8)

where *H*_*t*_ and *L*_*t*_ are the daily high and low prices, respectively. This estimator has an advantage over that based only on the closing prices because it uses information about the price changes during the day. As it can be seen it is calculated separately for each day *t*. Finally, in our study, we use the measure:

$${\tilde{\sigma }}^{\left(p\right)}=\frac{\bar{\sigma_{1}^{\left(p\right)}}-\bar{\sigma_{0}^{\left(p\right)}}}{\bar{\sigma_{0}^{\left(p\right)}}},$$ (9)

where $\bar{\sigma_{0}^{\left(p\right)}}$ and $\bar{\sigma_{1}^{\left(p\right)}}$ are the mean values of the Parkinson estimator over the base and pandemic periods, respectively. The value of ${\tilde{\sigma }}^{\left(p\right)}$ is the relative change in mean daily variability as measured by the Parkinson estimator.

The third measure of volatility stability is based on the range, that is, the difference between the maximum and minimum values in a given period. It is expressed by the formula:

$$\tilde{r}=\frac{r_{1}-\bar{r_{0}}}{\bar{r_{0}}},$$ (10)

where *r*_1_ is the range in the pandemic period, and $\bar{r_{0}}$ is the average value of the ranges of the base period composed of the same number of observations as the studied pandemic period. The range for the pre-pandemic period *r*_0_ for Period_2a, Period_2b and Period_2c was calculated as the range using a 1-day rolling window of two weeks, one month, and three months, respectively. This method of calculations allowed to use ranges calculated on the basis of the same periods as in the pandemic period in the calculations. In the case of Period_1 (which has the same length as the pre-pandemic period), *r*_0_ was calculated in the way, i.e., as the difference between the maximum and minimum price. The $\tilde{r}$ variable describes the relative change in volatility, measured by the range.

A measure based on the Relative Strength Index (RSI) was used to assess the attitude of investors. The RSI measures the magnitude of recent price changes to evaluate overbought or oversold conditions in the price of financial assets. The *n*-day RSI is calculated using the formula:

$${RSI}_{t}=100(1-\frac{D_{t}}{D_{t}+U_{t}}),$$ (11)

where *U*_*t*_ is an average of *n* days’ up closes and *D*_*t*_ is an average of *n* days’ down closes. In the study we assumed *n* = 14. Ultimately, the following measure was used to test the RSI stability:

$$\tilde{RSI}=\bar{{RSI}_{1}}-\bar{{RSI}_{0}},$$ (12)

where $\bar{{RSI}_{0}}$ and $\bar{{RSI}_{1}}$ are the average values of the RSI index over the base and pandemic periods, respectively. The measure $\tilde{RSI}$ shows the difference in the overbought in the two periods compared.

In turn, the following measure was used to assess the stability of turnover:

$$\tilde{W}=\frac{\bar{W_{1}}-\bar{W_{0}}}{\bar{W_{0}}},$$ (13)

where $\bar{W_{0}}$ and $\bar{W_{1}}$ are the average values of the trading volume in the base and pandemic periods. It means that $\tilde{W}$ is a relative change in the average volume of trading.

It should be noted that from the point of view of the aim of the study, the important question concerning stability is the scale of changes, and not their direction. Therefore, the absolute values of the above measures were applied in the clustering analysis. Moreover, to ensure a balanced effect of all these measures on the grouping result, they have been normalized prior to clustering. Lack of normalization gives a greater impact on the result obtained by variables expressed in larger numbers. For this purpose, all the variables were normalized using the min-max scaling with the formula:

$$x\prime =\frac{x-min(x)}{\max\left(x\right)-min(x)}$$ (14)

which ensures that all the transformed variables are in the range [0, 1].

## 3. Results and discussion

The starting point for our study was computing the values of the six measures for all the investigated sub-indices. We present the results in Tables 2–5.

**Table 2:** Values of the proposed measures for evaluated sub-indices–Period_1.

Index	$\tilde{P}$	$\tilde{\sigma }$	${\tilde{\sigma }}^{\left(p\right)}$	$\tilde{r}$	$\tilde{RSI}$	$\tilde{W}$
WIG-automobiles&parts	-0.065	2.424	1.111	2.994	9.692	0.820
WIG-banking	-0.298	1.620	1.063	1.417	4.509	0.336
WIG-chemical	-0.169	0.740	0.419	0.173	9.103	0.676
WIG-clothes	-0.242	1.798	1.054	2.361	-5.161	3.409
WIG-construction	0.171	0.957	0.697	0.916	4.260	1.191
WIG-energy	-0.222	0.885	0.566	1.440	4.813	1.120
WIG-food	-0.035	1.700	0.936	0.794	11.492	1.302
WIG.GAMES	0.503	1.701	1.134	3.467	6.084	0.957
WIG-IT	0.220	0.836	0.492	1.297	2.210	0.619
WIG-media	0.017	2.047	0.898	3.731	5.070	1.294
WIG-mining	-0.107	0.730	0.483	0.757	4.192	0.527
WIG-oil&gas	-0.278	0.777	0.684	0.838	-0.842	0.372
WIG-pharmaceutical	-0.224	1.518	0.966	1.517	2.068	20.115
WIG-real estate	-0.042	1.839	1.031	2.094	-7.984	0.279
WIGtech	0.185	2.142	0.904	2.511	6.049	0.583
WIG-telecom	-0.020	0.626	0.386	0.897	0.827	0.341

**Table 3:** Values of the proposed measures for evaluated sub-indices–Period_2a.

Index	$\tilde{P}$	$\tilde{\sigma }$	${\tilde{\sigma }}^{\left(p\right)}$	$\tilde{r}$	$\tilde{RSI}$	$\tilde{W}$
WIG-automobiles&parts	-0.281	4.917	3.032	2.474	-26.366	1.004
WIG-banking	-0.409	4.756	3.807	0.217	-20.115	0.476
WIG-chemical	-0.396	2.400	2.158	0.849	-11.273	1.482
WIG-clothes	-0.487	5.132	4.324	0.785	-31.538	5.834
WIG-construction	-0.076	1.780	2.300	0.106	-32.035	1.452
WIG-energy	-0.433	2.756	2.514	0.887	-13.694	2.683
WIG-food	-0.197	5.514	3.355	1.908	-13.104	5.268
WIG.GAMES	0.108	5.203	4.075	5.544	-20.728	2.761
WIG-IT	0.029	2.818	2.471	1.665	-26.895	1.315
WIG-media	-0.188	5.367	2.854	3.074	-27.453	0.862
WIG-mining	-0.390	2.457	2.262	0.389	-19.972	1.228
WIG-oil&gas	-0.472	1.807	2.631	0.834	-20.017	1.456
WIG-pharmaceutical	-0.445	4.333	3.724	1.866	-19.989	19.501
WIG-real estate	-0.142	2.910	2.924	4.772	-38.009	0.838
WIGtech	-0.029	6.585	4.193	5.339	-23.133	1.769
WIG-telecom	-0.134	2.568	2.079	2.255	-15.542	1.270

**Table 4:** Values of the proposed measures for evaluated sub-indices–Period_2b.

Index	$\tilde{P}$	$\tilde{\sigma }$	${\tilde{\sigma }}^{\left(p\right)}$	$\tilde{r}$	$\tilde{RSI}$	$\tilde{W}$
WIG-automobiles&parts	-0.275	4.125	2.124	1.492	-14.341	0.596
WIG-banking	-0.414	3.568	2.766	-0.170	-9.803	0.381
WIG-chemical	-0.359	1.636	1.370	0.997	6.765	1.360
WIG-clothes	-0.470	3.760	2.902	0.608	-15.880	6.862
WIG-construction	-0.049	1.612	1.771	0.651	-15.959	0.969
WIG-energy	-0.404	1.765	1.419	0.421	1.908	1.917
WIG-food	-0.169	3.978	2.557	2.192	2.824	2.936
WIG.GAMES	0.199	3.593	2.552	5.098	-3.537	1.778
WIG-IT	0.056	1.865	1.503	1.667	-13.851	0.862
WIG-media	-0.185	3.905	1.694	2.576	-15.338	0.766
WIG-mining	-0.351	1.660	1.381	0.503	-3.165	0.808
WIG-oil&gas	-0.412	1.339	1.913	1.302	3.271	1.353
WIG-pharmaceutical	-0.385	3.547	2.892	2.393	-3.152	41.669
WIG-real estate	-0.169	2.523	2.126	2.817	-34.709	0.487
WIGtech	0.015	4.553	2.587	4.894	-7.367	1.142
WIG-telecom	-0.112	1.726	1.324	1.812	-3.581	0.795

**Table 5:** Values of the proposed measures for evaluated sub-indices–Period_2c.

Index	$\tilde{P}$	$\tilde{\sigma }$	${\tilde{\sigma }}^{\left(p\right)}$	$\tilde{r}$	$\tilde{RSI}$	$\tilde{W}$
WIG-automobiles&parts	-0.171	3.077	1.313	1.894	5.125	0.544
WIG-banking	-0.436	2.212	1.699	0.054	3.056	0.739
WIG-chemical	-0.199	0.924	0.615	0.899	21.287	0.930
WIG-clothes	-0.366	2.370	1.674	1.775	5.358	5.651
WIG-construction	0.124	1.122	1.022	2.431	9.188	1.595
WIG-energy	-0.332	1.044	0.683	1.743	14.187	1.367
WIG-food	-0.091	2.357	1.392	1.390	14.137	1.735
WIG.GAMES	0.534	2.042	1.368	7.000	7.713	0.913
WIG-IT	0.209	1.103	0.815	3.124	2.895	0.793
WIG-media	-0.091	2.464	1.138	3.458	3.618	1.338
WIG-mining	-0.208	0.943	0.734	0.859	11.506	0.546
WIG-oil&gas	-0.334	0.790	0.912	0.759	10.466	0.581
WIG-pharmaceutical	-0.329	1.847	1.151	1.324	3.786	26.419
WIG-real estate	-0.130	2.187	1.447	2.434	-10.882	0.155
WIGtech	0.172	2.574	1.249	4.282	7.151	0.669
WIG-telecom	-0.061	0.865	0.626	0.530	2.449	0.472

When analyzing the obtained results, it is worth paying attention to the fact that in the relatively unfavorable price conditions prevailing at the outbreak of the pandemic, a positive measure of a profitability change was obtained for selected sectors. In particular, the sector of computer games producers (WIG.GAMES) and IT (WIG-IT) showed a positive change in pricings independently on the time frame of analysis. The technology industry (WIGtech) recorded positive change except for the shortest period of analysis (Period_2a) and the construction industry (WIG-construction) as well as media (WIG-media) increased their value in the medium term. All other sectors reported a profitability decrease. In case of variability measures ($\tilde{\sigma },\;{\tilde{\sigma }}^{\left(p\right)}$ and $\tilde{r}$) positive values were recorded in all analyzed periods for all investigated indices, except for a single record of the $\tilde{r}$ measure noted for the banking sector in the short-term (Period_2b). Such results clearly indicate increased volatility of valuation of WSE companies after the outbreak of COVID-19. Looking at the aforementioned parameter $\tilde{r}$, one should underline the positive outlier values for the computer games sector and the technology sector in all the analyzed periods, except Period_1. For the RSI variable, with some exceptions, one could observe negative values for all the sectors in the short term (Period_2a and Period_2b) and positive values in the medium term (Period_1 and Period_2c). This may point that the short period was characterized by a sell-off of shares and the medium by their buyout. The real estate sector (WIG-real estate) turned out to be the worst, recording the lowest and negative values in all the analyzed periods. The last variable $\tilde{W}$ is characterized by positive values for all the sectors and all the investigation periods. An interesting result was obtained is scope of trading volume of the pharmaceutical sector (WIG-pharmaceutical), and to the lesser extent the clothing sector (WIG-clothes), for which $\tilde{W}$ is significantly higher comparing to all other sectors in all the four periods of analysis.

After calculating the aforementioned six variables for all the sectors and for all the analyzed periods, we carried the clustering process on the basis of all these variables. In our study, we considered the number of clusters *K* = 2,3…,10 for both the *K*-means and Ward methods. To select the appropriate number of clusters the criterion of maximizing the silhouette coefficient was adopted. All calculations were made using own computer codes written in Python using the Scikit-learn [94] and the Yellowbrick [95] libraries.

Table 6 presents the obtained silhouette coefficients for the *K*-means and Ward methods for considered variants of the pandemic period. Values in the parentheses indicate the number of identified clusters.

**Table 6:** Silhouette coefficients for all six variables.

Pandemic period	Ward method	*K*-means method
Period_1	0.430 (3)	0.417 (2)
Period_2a	0.390 (8)	0.390 (8)
Period_2b	0.415 (7)	0.415 (7)
Period_2c	0.369 (5)	0.369 (5)

As it can be seen, in each case the obtained values are less than 0.5, which proves poor clustering. Therefore, an attempt was made to improve the quality of clustering, which consisted in limiting the set of diagnostic variables. Among the six variables initially accepted for the study, three refer to the same aspect–the volatility of quotations. As a result, there is a high probability that they may have a similar information load, which may not be favorable from the point of view of clustering quality. For this reason, it was decided to consider three variants of sets consisting of four diagnostic variables: $\tilde{P},\;\tilde{RSI},$ $\tilde{W}$ and, successively, only one of the measures of volatility stability: $\tilde{\sigma },\;{\tilde{\sigma }}^{\left(p\right)}$ and $\tilde{r}$. However, the study carried out on these three variants also yielded the silhouette coefficients of still less than 0.5 (results available on request).

Our further analysis showed that the reason for the low quality of the obtained clustering is the variable $\tilde{RSI}$. For this reason, it was rejected from the group of diagnostic variables. Finally, three variants of sets of variables were considered, consisting of three variables: $\tilde{P},\;\tilde{W}$ and, successively, one of the measures: $\tilde{\sigma },\;{\tilde{\sigma }}^{\left(p\right)}$ and $\tilde{r}$, with the best results being obtained using variable $\tilde{\sigma }$ as the measure of volatility stability. Table 7 presents the obtained silhouette coefficients for the *K*-means and Ward methods for considered variants of the pandemic period. Values in the parentheses indicate the number of identified clusters.

**Table 7:** Silhouette coefficients for three variables: $\tilde{P},\;\tilde{\sigma },\;\tilde{W}$.

Pandemic period	Ward method	*K*-means method
Period_1	0.509 (4)	0.509 (4)
Period_2a	0.512 (5)	0.512 (5)
Period_2b	0.601 (5)	0.601 (5)
Period_2c	0.572 (4)	0.572 (4)

Both *K*-means and Ward methods indicated the same division into groups in the case of the analyzed indices. In Period_1 and Period_2c, the highest silhouette coefficients occur when the indices are divided into 4 groups. However, in Period_2a and Period_2b, the division into 5 groups was optimal.

To assess the robustness of the results, we additionally employed the elbow method as the alternative tool for determining the optimal number of clusters [96–98]. The idea of this method is to calculate the within-cluster sum of squares (see Eq (1)) for different number of clusters (denoted by *SSE*(*K*)) and to plot a line chart of *SSE*(*K*) against the number of *K*. It can be easily seen that *SSE*(*K*) tends to decrease toward 0 as *K* increases. According to the method, one must pick the *K* value for which the line chart starts to flatten out and forms an elbow. Such a situation means that adding another cluster does not lead to much better clustering.

**Fig 2:**
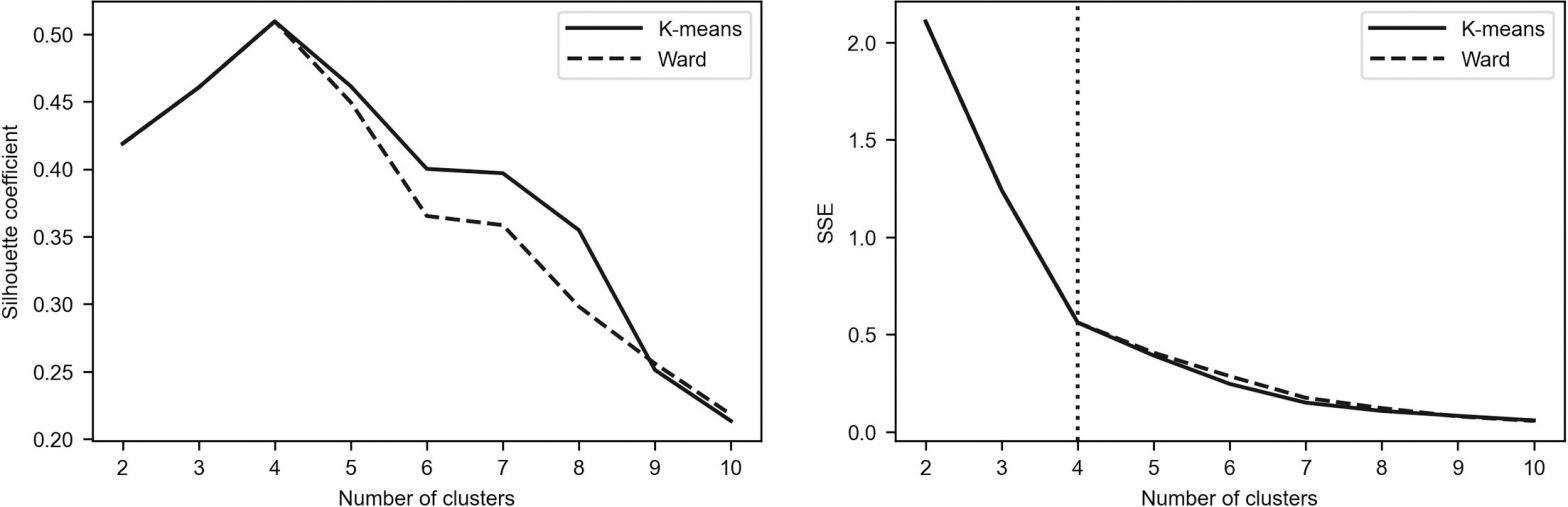
Silhouette coefficients and elbow curve–Period_1.

**Fig 3:**
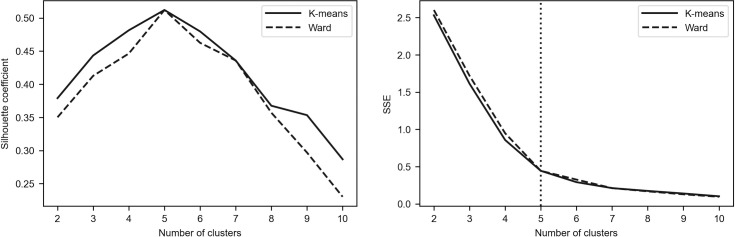
Silhouette coefficients and elbow curve–Period_2a.

**Fig 4:**
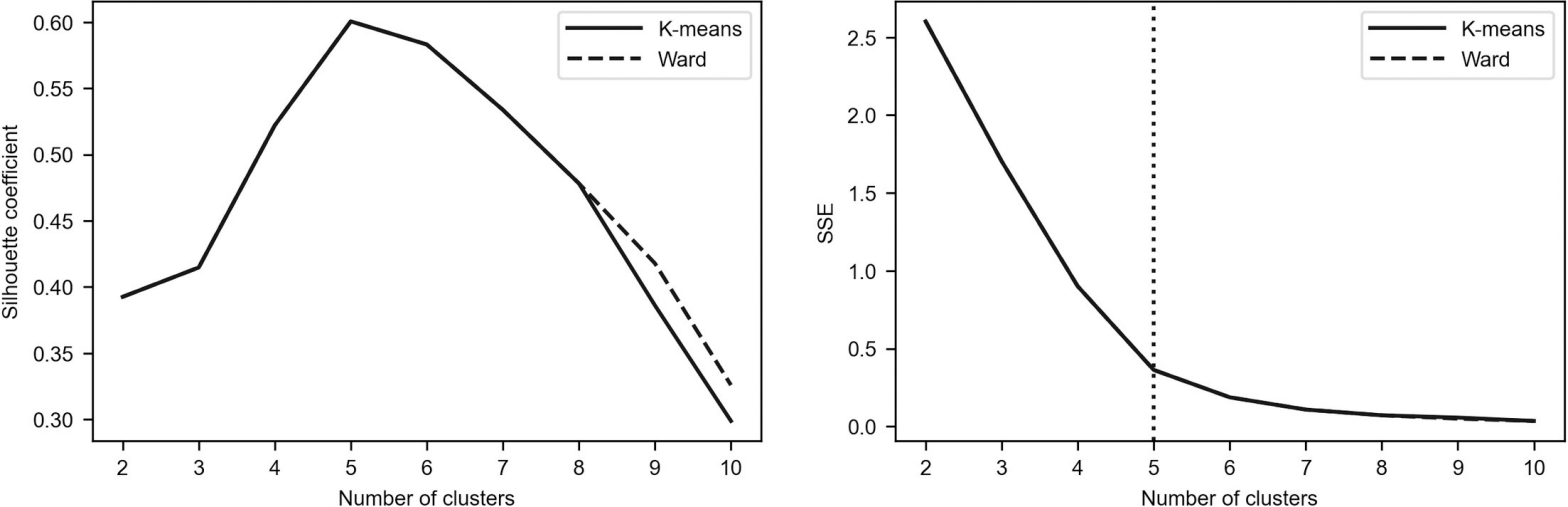
Silhouette coefficients and elbow curve–Period_2b.

**Fig 5:**
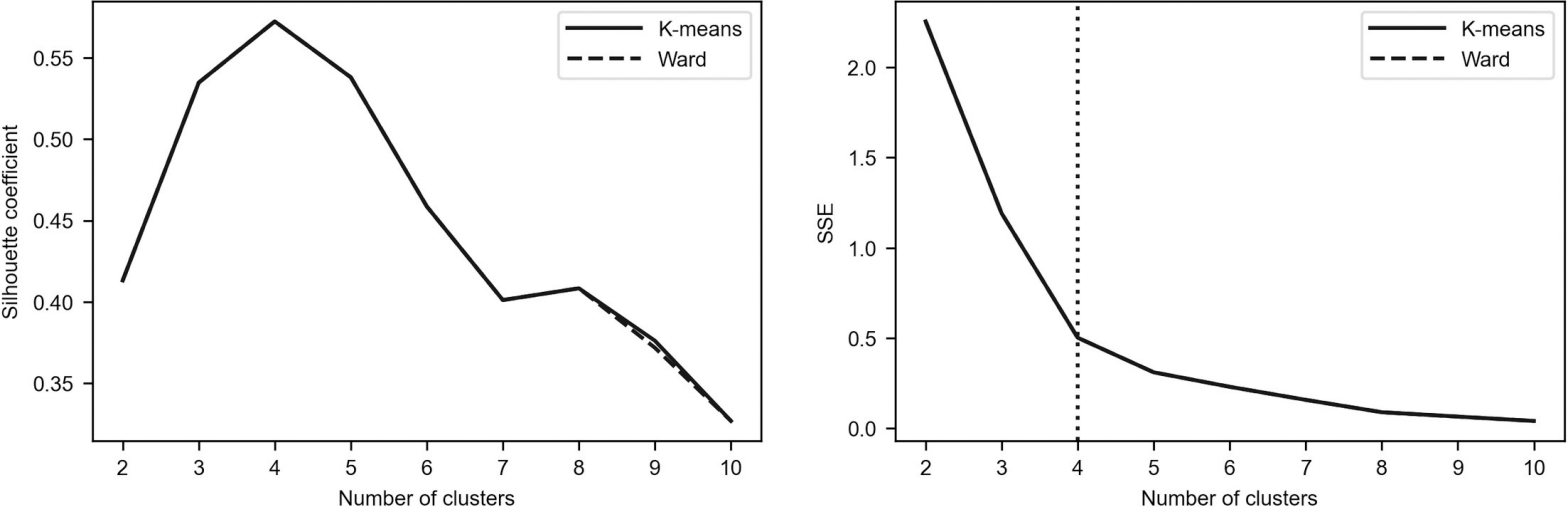
Silhouette coefficients and elbow curve–Period_2c.

Figs 2–5 depict the silhouette coefficients $\bar{s}(K)$ and the values of *SSE*(*K*) for *K* = 2,3,…,10. The vertical dotted line in the elbow method plot indicates the optimal number of clusters which was determined by using the knee point detection algorithm. The knee point detection algorithm finds the point of maximum curvature, which in a well-behaved clustering problem also represents the pivot of the elbow curve (see Bengfort and Bilbro [95] and Satopaa et al. [99]). We found that for all the investigated periods the elbow method points towards the same clustering as the method based on the silhouette coefficients.

Table 8 presents the composition of the obtained clusters and the mean silhouette coefficient for each of them (i.e. the average of the silhouettes for the indices which constitute the cluster). The value of the mean silhouette coefficient depends on the homogeneity of the cluster and its separation from the other ones.

**Table 8:** Composition of clusters in the investigated periods and their mean silhouette coefficients.

Cluster	Indices	Mean silhouette coefficient
Period_1		
Cluster_1	WIG-automobiles&parts, WIG-food, WIG-media,	0.532
	WIG-real estate, WIGtech	
Cluster_2	WIG-banking, WIG-clothes, WIG.GAMES	0.317
Cluster_3	WIG-chemical, WIG-construction, WIG-energy, WIG-IT,	0.649
	WIG-mining, WIG-oil&gas, WIG-telecom	
Cluster_4	WIG-pharmaceutical	0
Period_2a		
Cluster_1	WIG-food, WIG.GAMES, WIG-media, WIGtech	0.456
Cluster_2	WIG-automobiles&parts, WIG-banking, WIG-clothes	0.236
Cluster_3	WIG-chemical, WIG-energy, WIG-mining, WIG-oil&gas	0.736
Cluster_4	WIG-pharmaceutical	0
Cluster_5	WIG-construction, WIG-IT, WIG-real estate, WIG-telecom	0.679
Period_2b		
Cluster_1	WIG-automobiles&parts, WIG-food, WIG.GAMES,	0.503
	WIG-media, WIGtech	
Cluster_2	WIG-banking, WIG-clothes	0.664
Cluster_3	WIG-chemical, WIG-energy, WIG-mining, WIG-oil&gas	0.814
Cluster_4	WIG-pharmaceutical	0
Cluster_5	WIG-construction, WIG-IT, WIG-real estate, WIG-telecom	0.627
Period_2c		
Cluster_1	WIG-automobiles&parts, WIG-food, WIG-media,	0.667
	WIG-real estate, WIGtech	
Cluster_2	WIG-banking, WIG-clothes, WIG.GAMES	0.553
Cluster_3	WIG-chemical, WIG-construction, WIG-energy, WIG-IT,	0.595
	WIG-mining, WIG-oil&gas, WIG-telecom	
Cluster_4	WIG-pharmaceutical	0

Our classification of the indices representing individual sectors into the clusters partially coincided with the changes in the fundamentals of income generation and the development perspectives of such sectors in terms of the pandemic. Industries that turned out to be particularly susceptible to the business cycle and reduced activity due to lockdowns are metals, energy, machinery and equipment, chemistry or automotive manufacturers. On the other hand, there are computer & telecom, pharmaceuticals, software and IT, agri-food or construction, which supposed to be less susceptible to lockdowns [100]. When looking at such classification, we may point that in our study sectors such as WIG-chemical, WIG-energy, WIG-mining, WIG-oil&gas were classified within a single cluster (Cluster_3). Such clustering would correspond to a similar fundamental susceptibility of the companies from those, mainly traditional industries, to changes in the economic conditions caused by the pandemic. A similar effect was noticed in case of Cluster_5 including WIG-construction, WIG-IT, WIG-real estate, and WIG-telecom, for which lockdowns did not cause fundamental limitation of activity of companies. Both classifications became apparent only in the short term (Period_2a and Period_2b). In the longer time horizons, clustering put together sectors with different fundamental susceptibility to changes in the output during the pandemic, e.g. WIG-banking, WIG-clothes, WIG.GAMES (Cluster_ 2) or WIG-chemical, WIG-construction, WIG-energy, WIG -IT, WIG-mining, WIG-oil&gas, WIG-telecom (Cluster_3).

To evaluate in more details the obtained clusters, we analyzed the mean silhouette coefficient for each cluster (see Table 8). We found that clusters are generally characterized by a different degree of homogeneity. The highest differentiation between coefficients was observed in the shortest time horizon (Period_2a), and the lowest in the medium term (Period _2c). Cluster_3 in three out of four periods of analysis (except Period_2c) was characterized by the highest homogeneity. Taking into account the structure of this cluster in all the investigated periods we can note the strongest relationship formed by the chemical-fuel-energy sectors (WIG-chemical, WIG-energy, WIG-mining, WIG-oil&gas). In turn, Cluster_2 stands out in terms of the lowest homogeneity, reaching the minimum silhouette coefficient in the three out of four periods (except Period_2b). Cluster_4 always consisted of only one element, regardless of the period, that is why its coefficient equals 0.

The clustering results over four selected pandemic periods show that we are able to identify five groups of indices (proximities in stability) in the short terms (Period_2a and Period_2b) and four in the medium terms (Period_1 and Period_2c). It is worth noting that the indices creating Cluster_5 in the short periods moved principally to Cluster_3 in the medium terms.

Taking into account Period_1, we found proximities between WIG-media, WIG-automobiles&parts, WIG-real estate, WIG-food and WIG-telecom, then between WIG-banking, WIG.GAMES, WIG-clothes, and finally between WIG-construction, WIG-chemical, WIG-energy, WIG-mining, WIG-oil&gas, WIG-telecom and WIG-IT. The pharmaceutical sector remained solely classified. Such clustering results are observed also in Period_2c, what confirms the medium-term proximity of performance of groups of indices independently on the choice of the starting point of the pandemic (January 7 or March 12). The different clustering result is observed for Period_2a and Period_2b versus Period_1 and Period_2c. The bigger number of clusters may confirm that in the short periods (Period_2a and Period_2b) performance of the indices was more diversified.

According to the obtained clusters in the different pandemic periods, we found that some industries keep their proximities in all of the investigated periods. In Cluster_1 there were: WIG-media, WIG-food, WIG-telecom, in Cluster_2: WIG-banking, WIG-clothes and in Cluster_3: WIG-chemical, WIG-energy, WIG-mining, WIG-oil&gas. Cluster_4, consisting solely of WIG-pharmaceutical, also remained unchanged.

**Fig 6:**
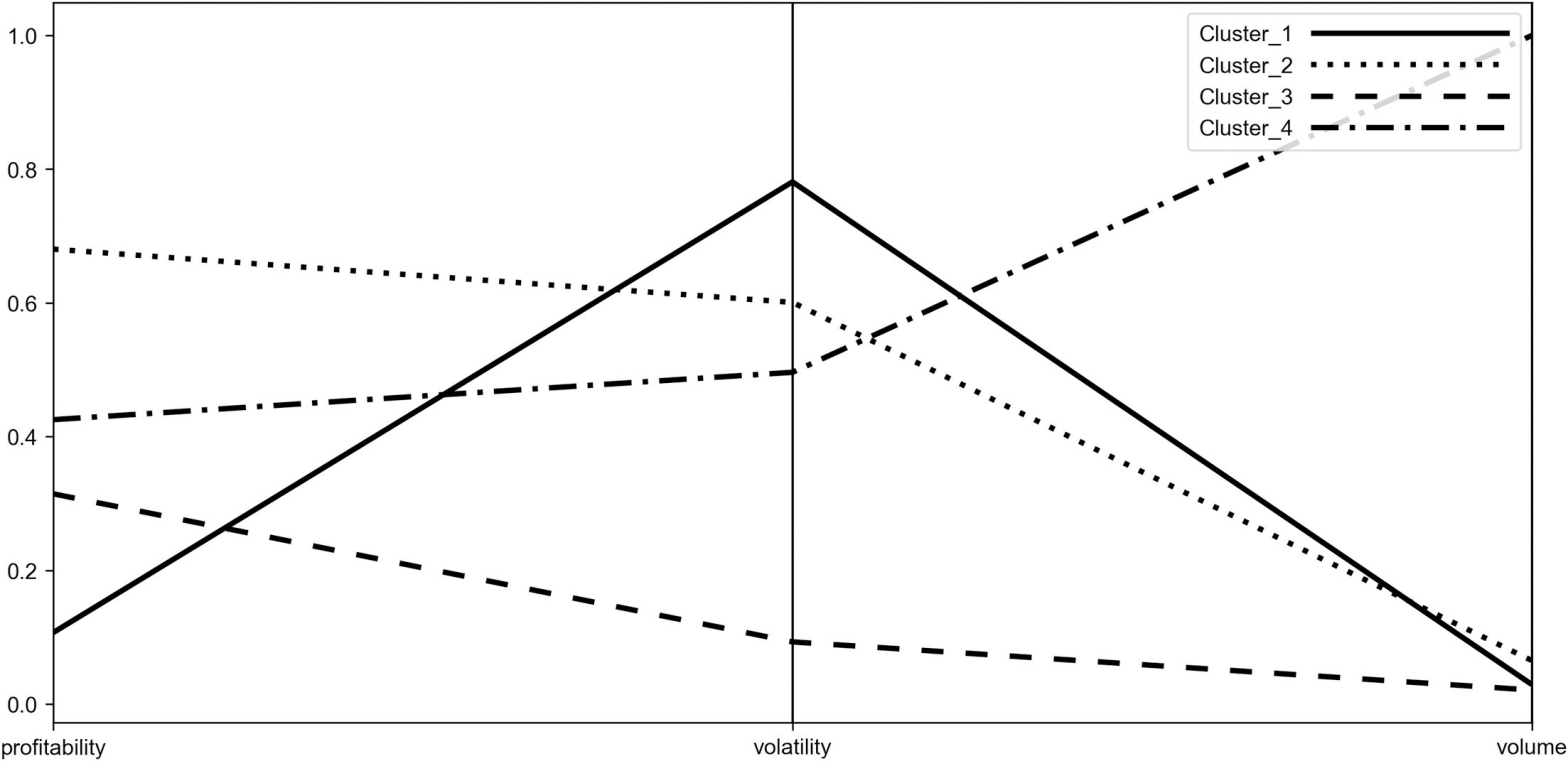
Parallel coordinate plot for centroids–Period_1.

**Fig 7:**
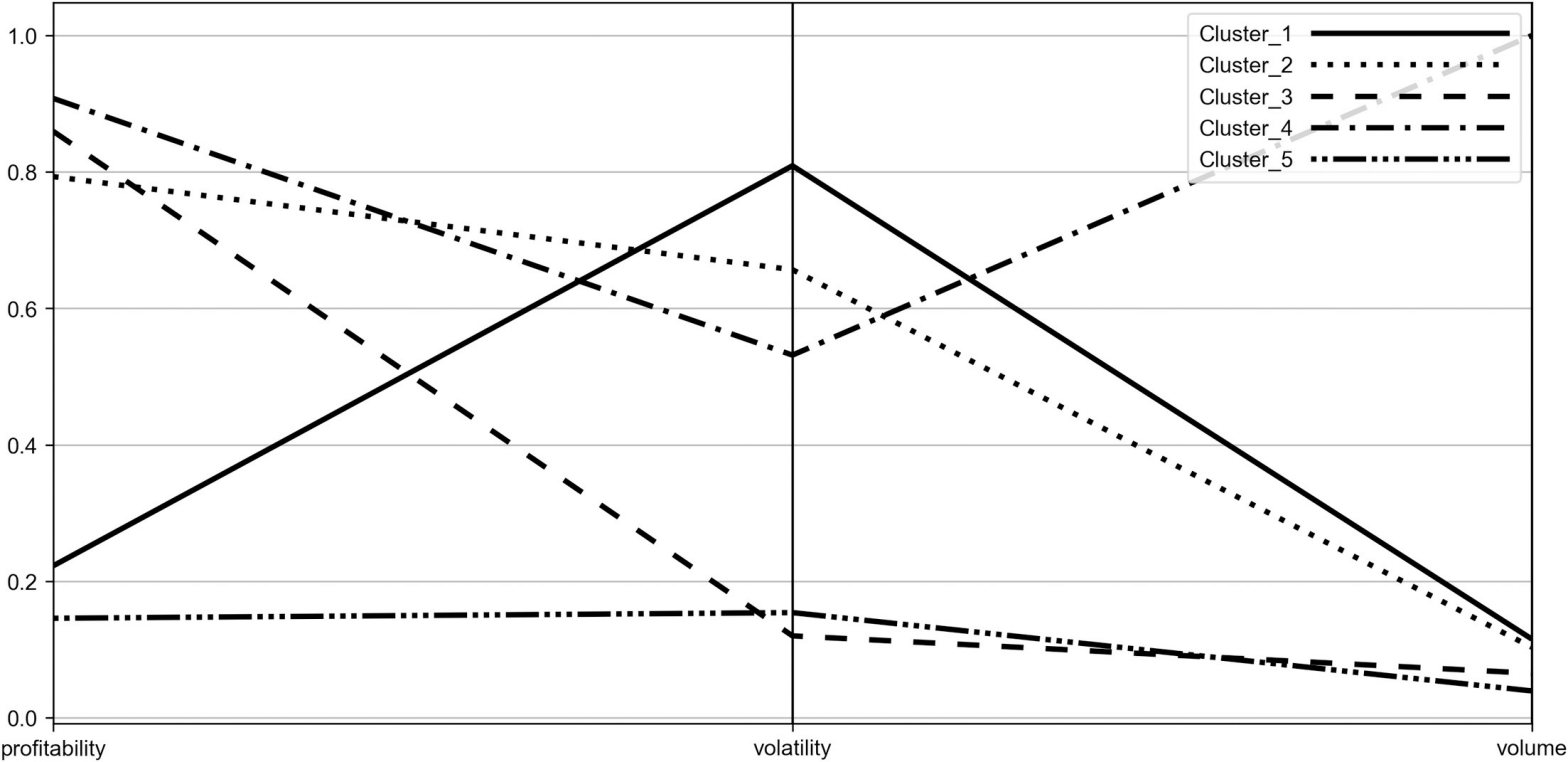
Parallel coordinate plot for centroids–Period_2a.

**Fig 8:**
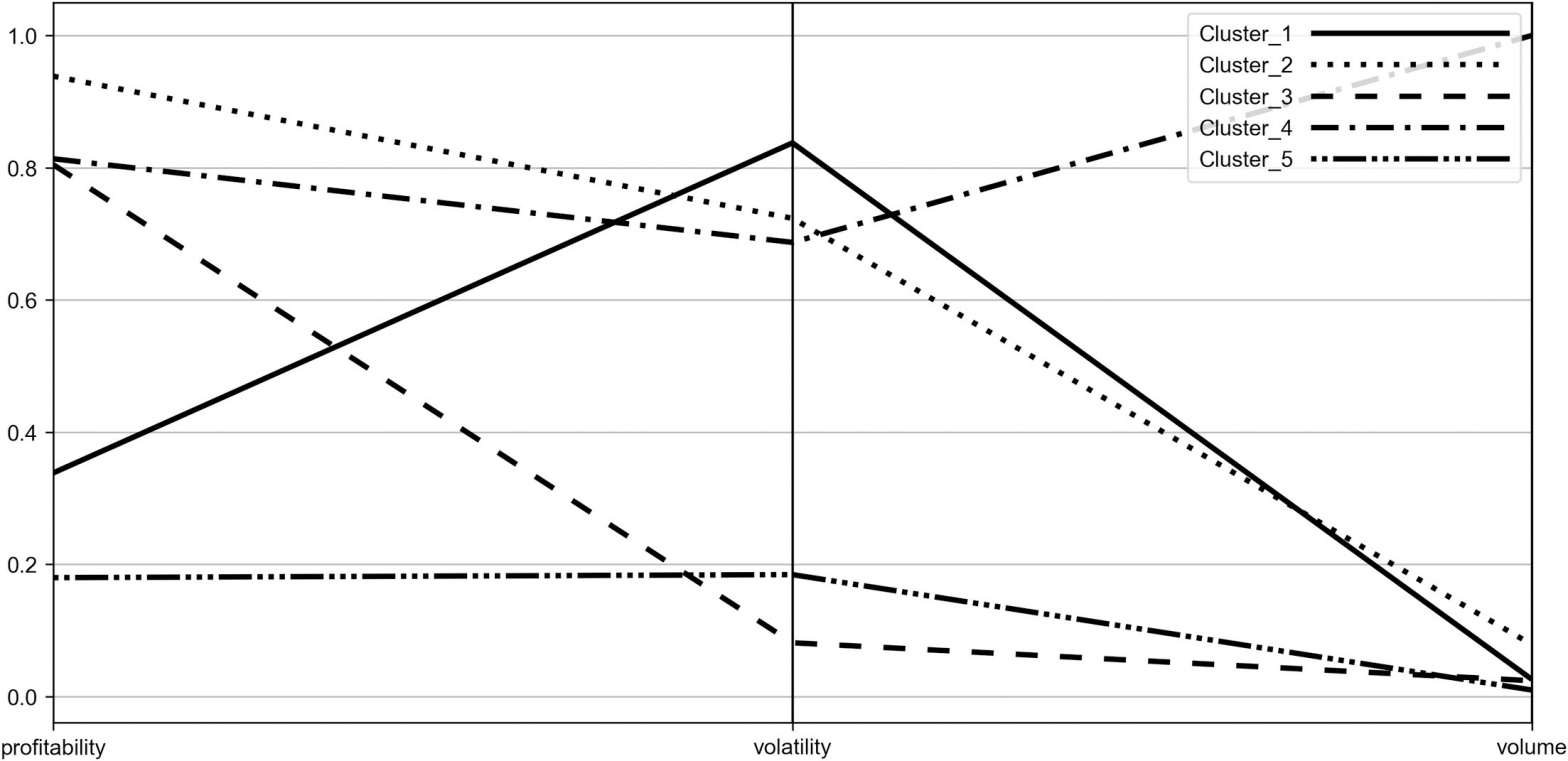
Parallel coordinate plot for centroids–Period_2b.

**Fig 9:**
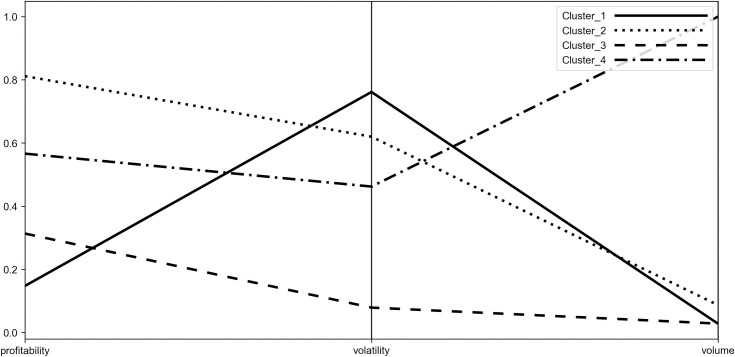
Parallel coordinate plot for centroids–Period_2c.

To characterize the obtained clusters more deeply and to identify their stability origins, the coordinates of their centroids were visualized with the use of parallel coordinates plot (see Figs 6–9). Such visualization shows the mean values of $\tilde{P},\;\tilde{\sigma },\;\tilde{W}$ for each group, therefore it allows to compare obtained clusters accordingly to the investigated diagnostic variables.

When analyzing the figures, one can find that the obtained clusters have different stability origins, i.e. they vary from each other in terms of the investigated indicators of stability. One can see that there is a large disproportion between clusters considering change of trading volume with the distant result noted by Cluster_4 (WIG-pharmaceutical) in all the investigated periods. In case of volatility, Cluster_3 (WIG-chemical, WIG-mining, WIG-oil&gas and WIG-energy) clearly distinguishes from other clusters, presenting the highest stability. Elevated stability in scope of volatility is also visible in the short period for Cluster_5 (WIG-construction, WIG-IT, WIG-real estate, WIG-telecom), which consists of indices constituting Cluster_3 in the medium periods. In case of profitability the clusters present dispersed results in the medium terms (Period_1 and Period_2c) and more polarized in the short terms (Period_2a and Period_2b). Profitability is also the factor that clearly differentiates Cluster_5 from Cluster_3, justifying the existence of the former in the short periods. One should also note that Cluster_3 in the medium term and Cluster_5 in the short term are characterized by the presence of sectors representing both positive as well as negative change in profitability. Nonetheless, their similarity can be found according to the absolute value of the profitability measure.

The results show that none of the distinguished clusters, and hence the indices included in the cluster, can be considered as the most or the least stable accordingly to all the investigated variables. However, we can draw some general conclusions about stability of the investigated indices, which we present underneath.

When evaluating stability from the short-term perspective, the entities from the WIG-construction, WIG-real estate, WIG-IT and WIG-telecom included in Cluster_5 can be viewed as the most stable in scope of trading volume and profitability. Taking into account the volatility, the most resilient to changes were companies from WIG-chemical, WIG-mining, WIG-oil&gas and WIG-energy representing Cluster_3, however Cluster 5 was also presenting a fair level of stability, with the highest proximity to Cluster_3.

In the medium term, the most stable according to volume and volatility were companies from Cluster_3 (WIG-chemical, WIG-mining, WIG-oil&gas and WIG-energy). Such results may confirm their relative high stability in general during turmoil independently on the time frame. The mentioned sectors are represented primarily by large entities and their stability can be explained by the ownership structure, including identified large stable shareholders, strategic economic importance, and monopolistic power. Taking into account the stability of the profitability in the medium term, Cluster_1 (WIG-media, WIGtech, WIG-food, WIG-automobiles&parts) was the leader.

When analyzing the stability of sectors regardless of the periods length, one should also pay attention to the performance of the industries belonging to Cluster_1, which show a relatively low change in volume and profitability, but at the same time are characterized by a high change in volatility. This phenomenon can be justified by the diversity and very different risk profile of the companies included in the indices that form Cluster_1. Moreover, when looking at the performance of the indices of Cluster_1, one may find that similar performance characterizes indices from Cluster_2, with the exception of profitability. As a rule, Cluster_2 presents a slightly higher change of volume and lower change of volatility than Cluster_1 but also vitally higher change in profitability. The relative similarities in terms of volume and volatility change between Cluster_1 and Cluster_2 can be observed altogether with instability of the composition of these clusters which is reflected in the process of interchanging some of their index components over time. It should be noted that the differences in change of profitability can be explained by the high variety of the sectors belonging to Cluster_2 and their very different potentials to generate profits in the pandemic. In Cluster_2 one may find both, the sectors recording a highly negative change in pricings, e.g. clothing with uncertain prospects for recovery due to closure of stores and lower demand on official wearing and banking industry suffering losses due to extremely low interest rates and abnormal loans defaults as well as the sector of computer games producers, showing positive profitability measure for all the investigated periods (see Tables 2–5). The linking factor for all of those sectors is a proximity of the absolute value of the change in profitability. A separate category is Cluster_4 represented by the pharmaceutical sector. This cluster shows a high change of volume against other sectors, relatively high change of volatility and profitability. In general, the overall stability of the pharmaceutical companies constituting Cluster_4 should be then evaluated as low. Separation of pharmaceutical industry results from other industries can be justified by the differentiated assessment of the future profits of the industry. Some part of the pharmaceutical companies may take advantage of the pandemic and get extraordinary profits while the other part may worsen their condition due to a decline in demand for pharmaceutical products not related to the pandemic.

## 4. Conclusions

The COVID-19 pandemic has a significant impact on the socio-economic situation of most countries in the world. It is undoubtedly a turning point in the activities of many sectors, as well as for the directions of development of the entire economies. In some industries it will undoubtedly cause a significant change in the business model or affect a structural change in the income and cost conditions. The consequences of changes and transformations in individual sectors are currently difficult to predict, as it is not known how long the pandemic will ultimately last and what its costs will be.

Our paper is devoted to the problem of stability of stock markets during the COVID-19 pandemic. Due to the low number of works related to CEE countries during the pandemic, we analyzed the Warsaw Stock Exchange, which is one of the most important stock markets in the CEE region. We assessed the stability of the behavior of different sectors of the economy represented by sector sub-indices and macro-indices of this market. In our study we applied two clustering methods: the *K-*means and the Ward techniques with the criterion of maximizing the silhouette coefficient. Due to the doubts concerning the turning point to be taken as the beginning of the pandemic and what period length of the pandemic is the most informative, we considered four time ranges. To perform the analysis, we proposed six indicators (diagnostic variables) describing stability in terms of profitability, volume, overbought/oversold conditions and volatility. We conclude that the use of all these variables resulted in a poor clustering results. However, we found that limiting the set of diagnostic variables to three aspects: profitability, volume and volatility leads to much better results. In this case the obtained results show that after the outbreak of the pandemic it was possible to observe on the market 5 clusters of sector indices in the short term (2 weeks and 1 month) and 4 in the medium term (3 and 6 months). The additional fifth cluster in the short term was extracted from Cluster_3 (indicated for the medium term). We found that the composition of the obtained clusters is quite stable, which means that many industries keep their proximities in all of the investigated periods. In Cluster_1 there were: WIG-media, WIG-food, WIG-telecom, in Cluster_2: WIG-banking, WIG-clothes and in Cluster_3: WIG-chemical, WIG-energy, WIG-mining, WIG-oil&gas. Cluster_4, consisting solely of WIG-pharmaceutical, also remained unchanged.

The results show that none of the distinguished clusters, and hence the indices included in the cluster, can be considered as the most or the least stable accordingly to all the investigated variables. For this reason, we additionally compared the obtained clusters in terms of their stability accordingly to separate indicators. Summarizing the results for the short periods, as the most unstable clusters we can point out Cluster_4, Cluster_2 and Cluster_3 –in terms of profitability, Cluster_1 –in terms of volatility and Cluster_4 –in terms of volume. On the other hand, the most stable clusters were Cluster_5 and Cluster_1 –in terms of profitability and Cluster_3 and Cluster_5 –in terms of volatility. In terms of volume all the investigated clusters except Cluster_4 were characterized by the similar level of stability. The most unstable clusters in the medium periods were Cluster_2 –in terms of profitability, Cluster_1 –in terms of volatility and Cluster_4 –in terms of volume. As the most stable clusters we can indicate Cluster_1 –in terms of profitability, Cluster_3 –in terms of volatility. Same as with short periods, in terms of volume all the investigated clusters, except Cluster_4, were characterized by the similar level of stability. Generally, we can conclude that Cluster_3 (in all periods) and Cluster_5 (in the short period) distinguishes from other clusters in terms of their overall stability, and that Cluster_4 can be considered the most unstable.

The results obtained from our research may bring several significant benefits to individual as well as institutional stock exchange investors. Determining the number of clusters and their compositions allows for better understanding of the behavior of industries and their companies in terms of external shocks, and thus for taking investment decisions that optimize the composition of the portfolio of securities. As our research characterizes the proximities in market behavior of multiple sectors, the investors may manage the investment risk in more effective way. By identifying industries that are slightly responsive to the crisis (most stable) or the strongly responsive (most unstable), investors can propose investment strategies focused on capital protection (defensive) or speculation (aggressive). Moreover, knowing the stability profile of individual sectors (according to profitability, volatility, turnover), investors can develop specific investment strategies within each cluster. Including this knowledge may also support more effective application of derivatives, such as futures or options, to manage investment portfolios.
